# Nanoscale Imaging of Kidney Glomeruli Using Expansion Pathology

**DOI:** 10.3389/fmed.2018.00322

**Published:** 2018-11-21

**Authors:** Octavian Bucur, Yongxin Zhao

**Affiliations:** ^1^Department of Pathology and Cancer Research Institute, Beth Israel Deaconess Medical Center, Harvard Medical School, Boston, MA, United States; ^2^Ludwig Center at Harvard Medical School, Boston, MA, United States; ^3^Department of Molecular Cell Biology, Institute of Biochemistry of the Romanian Academy, Bucharest, Romania; ^4^Department of Biological Sciences, Mellon College of Science, Carnegie Mellon University, Pittsburgh, PA, United States

**Keywords:** tissue expansion, nanoscopy, expansion microscopy, kidney glomerulus, imaging, immunostaining

## Abstract

Kidney glomerular diseases, such as the minimal change disease (MCD) and focal segmental glomerulosclerosis (FSGS), and other nephrotic syndromes, are typically diagnosed or confirmed via electron microscopy. Although optical microscopy has been a vital tool to examine clinical specimens for diagnoses in pathology for decades, the optical resolution is constricted by the physical diffraction limit of the optical microscope, which prevents high-resolution investigation of subcellular anatomy, such as of the podocyte tertiary foot processes. Here, we describe a simple, fast, and inexpensive protocol for nanoscale optical imaging of kidney glomeruli. The protocol is based on Expansion Pathology (ExPath), a new principle of microscopy that overcomes optical diffraction limit by chemically embedding specimens into a swellable polymer and physically expanding it homogenously prior to imaging. Our method uses only commercially available reagents, a conventional fluorescence microscope and it can be applied to both fixed-frozen or formalin-fixed paraffin embedded (FFPE) tissue sections. It requires minimal operative experience in a wet lab, optical microscopy and imaging processing. Finally, we also discuss challenges, limitations and prospective applications for ExPath-based imaging of glomeruli.

## Introduction

In pathology, examination of cellular structures and molecular composition using diffraction-limited microscopy has long been key to the diagnosis of a wide variety of pre-disease and disease states (1, 2).

Kidney podocytes and their foot processes are a key component of the ultrafiltration system in the glomerulus, where they comprise the filtration barrier together with endothelial cells and the glomerular basement membrane (GBM) (3, 4). Podocytes have unique anatomy, characterized by a cell body, major (primary and secondary) foot processes, and branches of minor (tertiary) foot processes that look like interdigitating fingers, attached to the GBM. This unique ultrafine structure functions as primary filter to allow water, solutes, and small proteins to pass through the capillary lumen into Bowman's space. It has been increasingly accepted that podocyte's function and structure are key factors defining the integrity of glomerular filtration barrier. Proteinuric kidney diseases are typically associated with various degrees of podocyte membrane remodeling (FP effacement and/or slit diaphragm (SD) disruption) driven by a rearrangement of the podocyte microfilament system, such as in the FSGS and MCD (4, 5). High-grade nephrotic-range proteinuria found in glomerular pathologies are preceded by ultrastructural changes in the FP morphology. In the case of MCD, that may be the only detectable anatomic abnormality. Due to the small size of the tertiary foot processes, these lesions cannot be observed by conventional diffraction-limited microscopy. Our current capability to assess their pathological alteration relies on electron microscopy (EM) (6, 7). However, EMs are expensive and are not a common imaging equipment in hospitals and even research labs. They are slow and difficult to use, requiring trained individuals for efficient operation. Other imaging technologies, such as the super-resolution optical microscopies, can overcome optical diffraction limit (8–11). However, they are also very complex, slow and expensive. therefore, not readily accessible in a clinical setting. Thus, there is a need for higher resolution microscopy that can work with current diffraction limited microscopes and can optically magnify renal specimens with nanoscale precision.

**Figure 1:**
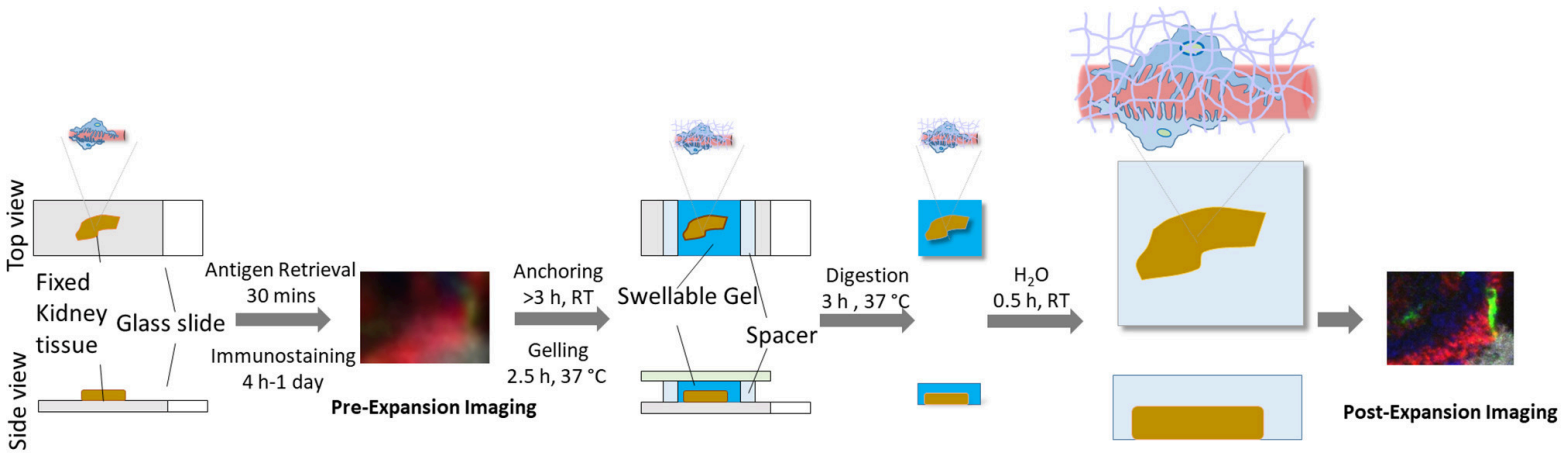
Schematic of ExPath workflow for kidney glomeruli imaging.

Here, we describe and discuss a protocol for the optical nanoscale imaging of kidney glomeruli, based on a novel super-resolution optical imaging technique, Expansion Pathology (ExPath) (12). ExPath is a variant of Expansion Microscopy (ExM) (13) specific for nanoscale imaging of clinical specimens. In contrast to conventional optical approaches that harness optics and hardware, ExPath overcomes diffraction limit by physical tissue expansion. ExPath comprises four key steps: (1) Chemically convert various formats of human tissue specimens to a state for sequential chemical modification and physical expansion; (2) Chemically modify the tissue and re-embed it into a water-swellable polymer synthesized *in situ*; (3) Remove unlabeled biomolecules and structural proteins using aggressive proteinase K treatment; (4) Physically expand the gelled tissue in pure water (Figure 1). ExPath protocol allows biomolecules that are covalently linked to the polymer chain within diffraction limited space to be isotropically separated from each other throughout the tissue by hydrogel expansion. Therefore, it facilities optical imaging with sub-diffraction limit resolution using only a conventional optical microscope. In addition, unlike the original ExM that requires customized oligo-conjugated antibodies (13), ExPath only requires commercially available reagents and minimal experience in optical imaging and wet-laboratory operation (12). With ExPath, the ultrafine structure of the tertiary podocyte foot processes is resolvable using conventional optical microscopes commonly found in many pathology laboratories (12), which may potentially foster novel optical diagnosis of MCD and other podocytopathies.

## Materials and equipment

The un-identified archival kidney pathological specimen used in Figure 5 was provided by the Brigham and Women's Hospital archives under the BWH IRB protocol #2011P002692 to Astrid Weins. Normal human kidney tissue specimens were purchased from US Biomax, Inc (cat. no. HuFPT072).

Reagents used in this protocol are listed in Table 1.

**Table 1:** Reagents used in this work.

**Name**	**Vendor**	**Catalog#**	**Comments**
Acetone	Fisher Scientific	S25904	used for fixation of fresh-frozen tissue
Formalin, 10% v/v	Electron Microscopy Sciences	15740	used for fixation of FFPE specimens
Milli-Q water		
PBS, 1 ×	Life Technologies	10010023
PBS, 10 ×	Thermo Fisher Scientific	AM9625
Xylene	Thermo Fisher Scientific	1330-20-7
Ethanol	Thermo Fisher Scientific)	64-17-5
Sodium citrate tribasic dihydrate	Sigma-Aldrich	C8532	for antigen retrieval solution preparation
MAXblock™ Blocking Medium	Active Motif	15252	Blocking buffer
MAXbind™ Staining Medium	Active Motif	15253	Staining buffer
MAXwash™ Washing Medium	Active Motif	15254	Washing buffer
DAPI, 1M	Thermo Fisher Scientific	62248
Acryloyl-X, SE	Life Technologies	A20770	10 mg/ml stock solution in DMSO, stored in a desiccated environment at −20°C
Sodium acrylate	Sigma-Aldrich	408220
Acrylamide	Sigma-Aldrich	A8887
N,N′-Methylenebisacrylamide	Sigma-Aldrich	M7279
Sodium chloride	Sigma-Aldrich	S6191
4-hydroxy-TEMPO (4-HT)	Sigma-Aldrich	176141
N,N,N′,N′-etramethylethylenediamine (TEMED)	Sigma-Aldrich	T9281
Ammonium persulfate (APS)	Sigma Aldrich	248614
Proteinase K, 800 units/ml (Molecular Biology Grade)	New England Biolabs	P8107S
Trizma® base	Sigma Aldrich	T1503
Ethylenediaminetetraacetic acid disodium salt dihydrate (EDTA)	Sigma-Aldrich	E4884
Primary antibodies	various	various	concentration varies with the antibody used
Secondary antibodies conjugated with fluorescent dyes	various	various	recommended fluorescent dyes for conjugation: Alexa Fluor 488, Alexa Fluor 546, Alexa Fluor 560, ATTO 647N, CF633, CF640R.

For convenience, we recommend users preparing the following stock solution prior to experiments:

1. **Acryloyl-X, SE (AcX) stock solution:** 10 mg/ml stock solution in DMSO. Store in a desiccated environment at −20°C.
2. **Sodium citrate solution:** 20 mM sodium citrate at pH 8.0.
3. **Monomer solution:** 8.6 g/100 ml sodium acrylate, 2.5 g/100 ml acrylamide, 0.1 g/100 ml N,N′-methylenebisacrylamide, 4 g/100 ml Sodium chloride, in PBS. Store the monomer solution mix at 4°C for up to 3 months or freeze at −20°C for long term storage.
4. **TEMED accelerator stock solution:** 0.1 g/ml TEMED in ddH_2_O.
5. **4-HT inhibitor stock solution:** 0.005 g/ml 4-HT in ddH_2_O.
6. **Ammonium persulfate (APS) initiator stock solution:** 0.1 g/ml APS made fresh for each experiment.
7. **Digestion buffer:** 50 mM Tris, 25 mM EDTA, 0.5% Triton X-100, 0.8 M NaCl, and then adjust pH to 8.0. Store the buffer as aliquots in the fridge at 4°C for up to 6 months.

**Microscope setup:** configure a four-channel microscope with appropriate excitation light sources and emission filters for fluorophores. In our case: DAPI, Alexa Fluor 488, Alexa Fluor 546 and ATTO 647N (Table 2). Optionally, a long working distance 40 × water immersion objective can be used for large volume wide-field and confocal imaging (examples: Nikon's CFI Apo Lambda S LWD 40 × WI and Zeiss's C-Apochromat 40 × /1.2 W Corr M27 lens).

**Table 2:** Example of filter sets combination for four color imaging.

**Excitation (nm)**	**Dichromatic (nm)**	**Emission (nm)**	**Example fluorophore**
325–375	400	435–485	DAPI
451–490	497	502–542	Alexa Fluor 488
532–588	595	604–679	Alexa Fluor 546
590–650	660	663–738	Atto 647N/CF640R

## Stepwise procedures

This protocol can be applied to both fixed frozen and FFPE kidney tissue sections. Note that the pre-expansion steps are similar to the ones from a conventional IF protocol, since the expansion is performed after immunostaining is completed. Thus, the immunostaining procedure described in this protocol can be replaced by users' customized immunostaining protocols. The basic steps are listed below (For troubleshooting, see Table 3):

**Table 3:** Potential problems with staining, distortion and expansion factor.

**Problem**	**Potential causes**	**Solution**
Low or absent fluorescence signal (pre-expansion)	1. Ineffective antigen retrieval; 2 Suboptimal immunostaining	Optimize antigen retrieval conditions and immunostaining parameters or change the antibody used.
Strong signals pre-expansion but low or absent fluorescence signals (post-expansion)	Fluorescent dyes may be bleached.	Use our recommended dyes for conjugation (Table 2); make sure the gel is on the right side before imaging; shorten the digestion time
Microscopic cracks or other distortion after expansion	Incomplete digestion	Optimize digestion parameters; our recommended concentration and incubation times work for all tissues that we've tried; however, there may be some others that require slightly different digestion parameters.
Expansion factor lower than 4	1. Incomplete wash with pure water. 2. Improper ratio of acrylate to acrylamide	1. Prolong wash with pure water 2. Check gel composition

### Specimen pre-processing

For fixed-frozen tissue slides, leave the slides at room temperature (RT) for 2–5 min, followed by washing with 1 × PBS (3 times, 5 min each washing, at RT).

For unfixed fresh-frozen slides, fix the tissue with a non-formalin fixative, such as with cold acetone (equilibrated at −20°C) for 10 min at −20°C, followed by drying at RT for 1 min and washing with 1 × PBS solution (3 times, 5 min each washing, at RT).

For FFPE tissue slides, bake the specimen at 60°C for 10 min, then deparaffinize the formalin-tissue sections by sequentially placing tissue sections in a series of solutions for 3–5 min each step at RT: 2 × xylene, 2 × 100% ethanol, 95% ethanol, 70% ethanol, 50% ethanol, and finally 1 × PBS.

### Heat induced antigen retrieval

For optimal immunostaining results, this step is needed regardless of the fixatives used. Immerse the specimens in 20 mM sodium citrate solution (pH 8, 100°C) in a heat-resistant container, and then transfer the whole covered container to a 60°C incubation chamber for 30 min (the temperature will slowly decrease to 60°C). Then remove the cover and let the container at RT for 5–10 min before proceeding with the following step.

### Immunostaining

Starting with this step, do not let the tissue sections dry out. Use a hydrophobic pen to encircle the tissue section on the glass slide and keep the solution on the tissue. Antibody concentration should be used as recommended by the manufacturer, or as empirically determined.

Treat the kidney tissue with MAXblock™ Blocking Medium, 1 h at 37°C for background reduction. Incubate with primary antibodies in MAXbind™ Staining Medium, at a concentration of 10 μg/ml (please check recommended concentration for each antibody used) overnight at 4°C, or 6–12 h at RT, depending on tissue thickness and the antibodies (if a mix of antibodies is used, make sure that they originate from different species, e.g., mouse, rat, rabbit, goat, chicken, guinea pig, or different subclasses for the same host species, e.g., IgG_1_, IgG_2b_, and IgG_3_ for mouse). After washing 3 times with MAXwash™ Washing Medium (each washing for 10 min), incubate with fluorescently labeled secondary antibody (antibodies) at a concentration of ~10 μg/ml in MAXbind™ Staining Medium for 2 h at RT. Thicker sections or specific antibodies may require increased incubation times.

### Pre-expansion imaging of specimens (optional)

Pre-expansion imaging is useful for calculating expansion factor and estimating post-expansion distortion. For imaging with conventional light microscopy, add ~50 μl of 1 × PBS on the tissue, then place a coverslip to cover the tissue slide (make sure no bubbles are present). Insert the slide into the microscope making sure that the coverslip/tissue side is facing the lens and image the regions of interest at desired magnification (Figures 4A,B). It is also recommended to image the region(s) of interest with the same objective (e.g., 40 × long distance objective) used to image later the expanded tissue. Specimens can be stored in PBS overnight or for a few days, at 4°C.

### Protein anchoring

Incubate kidney tissue slides with ~250 μl AcX, for 3 h at RT, at a concentration of 0.03–0.1 mg/ml (note that the concentration varies with the nature of the fixative used: 0.03 mg/ml for kidney specimens fixed with non-aldehyde fixatives, such as acetone or ethanol; 0.1 mg/ml for specimens fixed with aldehyde fixatives, such as formalin) in 1 × PBS. This step can also be performed overnight, at 4°C.

### *In situ* gelling

Prepare the gelling solution by mixing the following 4 solutions on ice in the following order: monomer solution (see above), TEMED accelerator solution (from the 10% stock solution, final concentration 0.2%, 1:50 dilution), 4-HT inhibitor solution (from the 0.5% stock solution, final concentration 0.01%, 1:50 dilution), ammonium persulfate (APS) initiator solution (from the 10% stock solution, final concentration 0.2%, 1:50 dilution).

**Figure 2:**
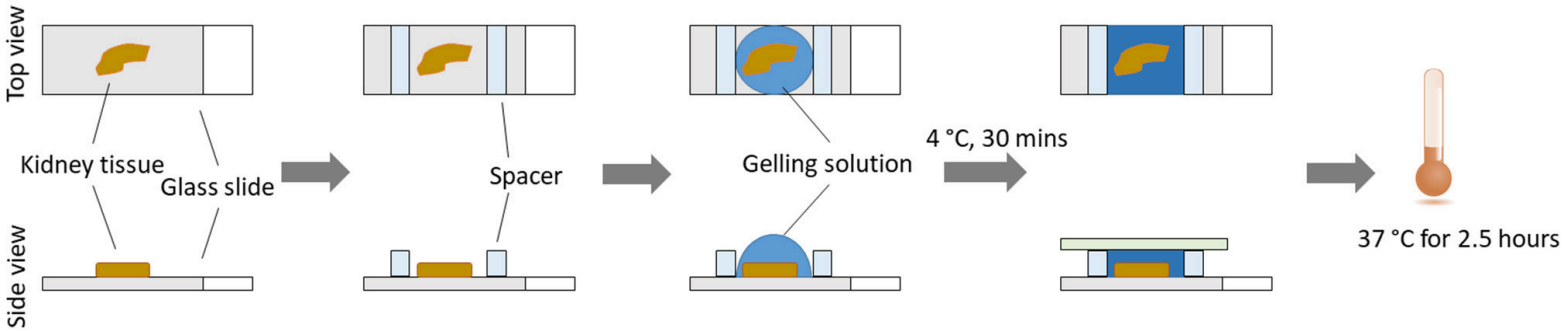
Steps for *in situ* gelling process.

Incubate the tissue slides with the gelling solution (~250 μl of gelling solution for each of the tissue slides) for 30 min at 4°C (4°C effectively slows down the free radical polymerization; 30 min incubation is sufficient for efficient diffusion into a 5 μm thick tissue section; lengthen the incubation time up to 1 h for thicker tissues). Replace with the same amount of fresh solution and ensemble the tissue slide into a gel chamber, by constructing a sandwich for the tissue section on the slide. Briefly, the tissue section from the slides is covered with a coverslip, with spacers on each side of the tissue section to prevent compression of the tissue slice. Spacers are made by cutting pieces of coverslips using a diamond knife. Make sure that there are no air bubbles. Incubate for at least 1.5 h at 37°C, in a humidified environment. The solution will be solidified (Figure 2).

### Homogenization

**Figure 3:**
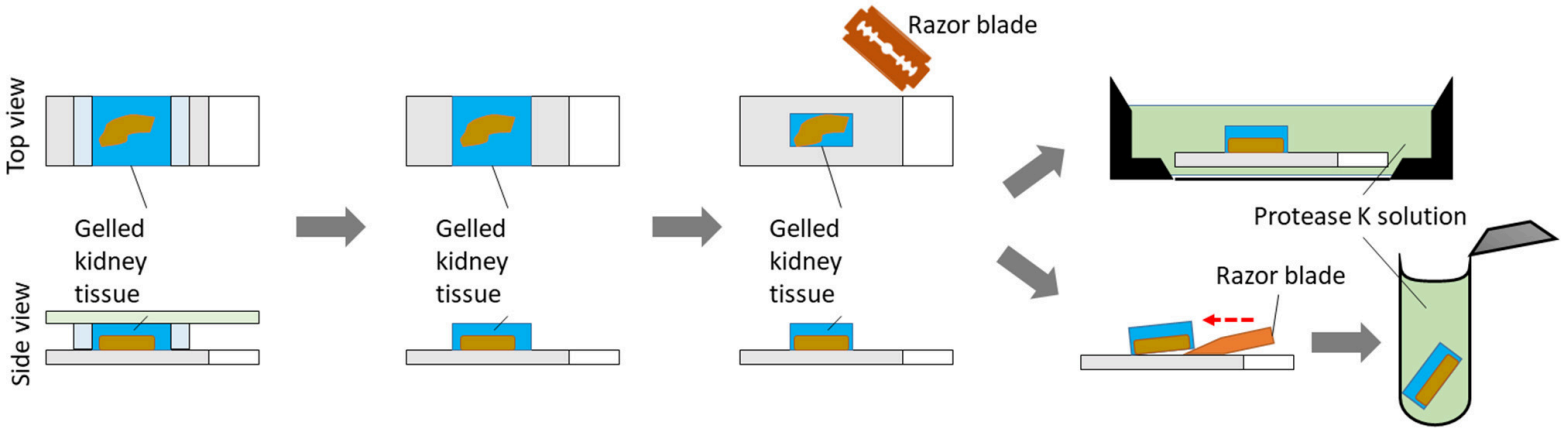
Steps for protease-K assisted homogenization process.

Remove the coverslip. Trim out blank gel regions and remove spacers. Place the gelled specimen in an appropriate container, such as a 4-well cell culture plate. Incubate the gel (still on the slide, fully submerged) with 3 ml of freshly prepared proteinase K digestion buffer for 3 h at 60°C. Normally the specimen will detach from the glass slide by itself after digestion. However, if needed, use a razor blade to gently remove the gel containing the tissue off the slide after digestion. Alternatively, tissue can be gently removed from the slide with a razor blade before incubation with proteinase K (Figure 3). Place the specimen in an appropriate container, such as a 6-well black-walled plate with transparent glass bottom, for the following steps.

### Nuclei staining

Wash the tissue slides once with 1 × PBS buffer for 10 min at RT and stain with 300 nM DAPI in PBS buffer for 5 min at RT. Wash again once with 1 × PBS for 10 min at RT.

### Visualize the tissue for right side positioning (optional)

**Figure 4:**
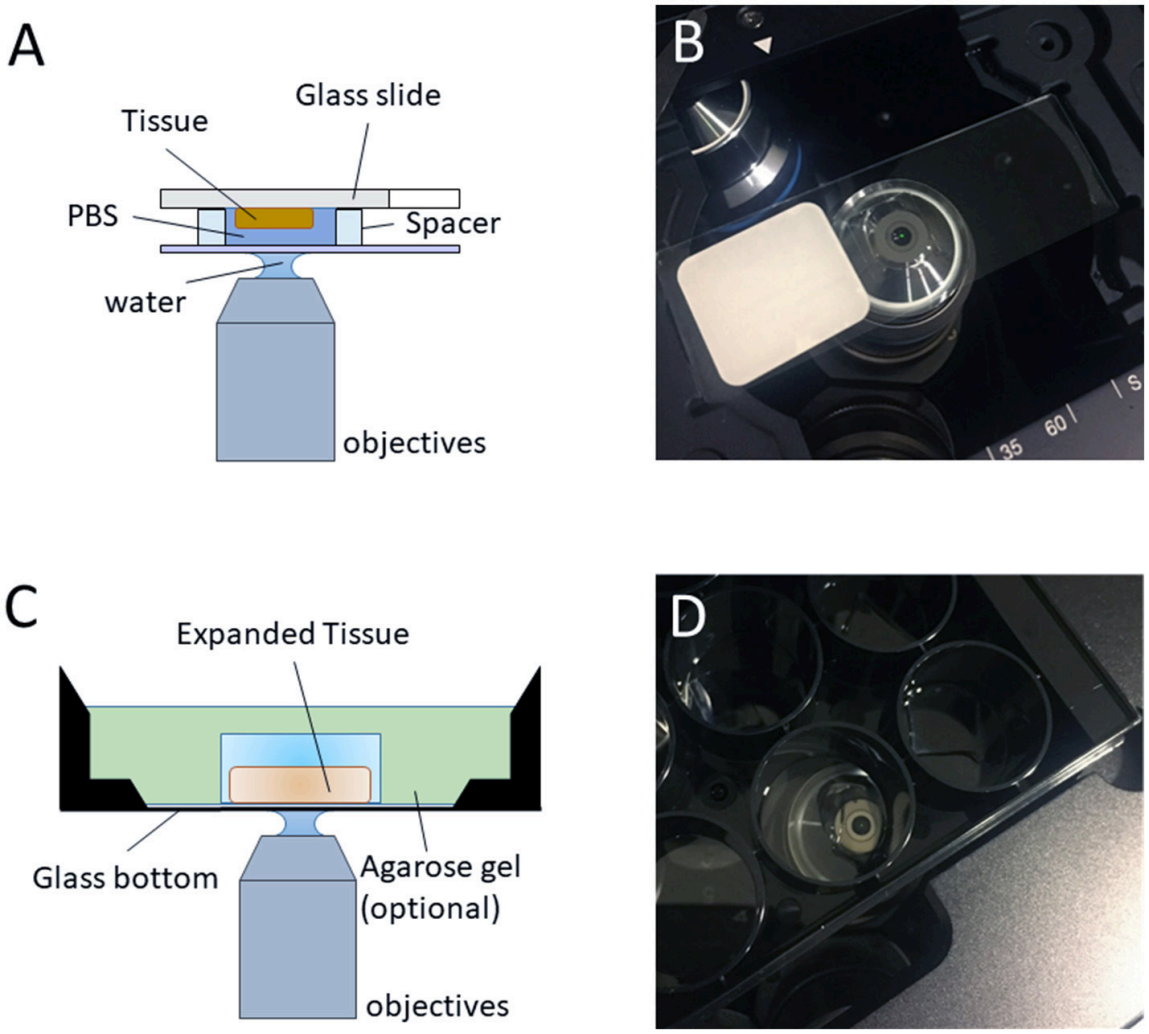
Imaging regular/expanded kidney tissue sections with inverted fluorescence microscope. **(A)** The configuration of the objective and the regular kidney specimen on a glass slide with a water-immersion objective (optional, air objective or oil objective can also be used for imaging) for pre-expansion imaging. **(B)** Photo of the typical setup on an inverted microscope, as depicted in **(A)**. **(C)** The configuration of the objective and the expanded sample on an inverted microscope with a water-immersion objective (optional, air objective or oil objective can also be used for imaging) for post-expansion imaging. **(D)** Photo of the typical setup on an inverted microscope, as depicted in **(C)**.

If using high-magnification objectives is needed, it is important to ensure the tissue side of the gel is facing the objective (Figures 4C,D). Otherwise, the tissue will be beyond the working distance of high-magnification objective. It is useful to image the specimen using a low-magnification objective in a fluorescence microscope and measure the z position when the sample is in focus. In this case, shrink the gelled tissue in a salt-containing solution, such as PBS, and then use a wetted paint brush to flip it in the solution. Handling gelled tissues without placing them in a salt-containing solution or handling fully-expanded tissues may result in specimen damage.

### Expansion

Wash the specimens with an excess volume of pure water (we usually use at least 10 folds of the final gel volume). Repeat 4 times for 5 min each time at RT. Expansion should reach a plateau after >3 washes. The specimen may have to be cut in smaller pieces before expansion, if the size of the chamber is not adequate.

Note that tissue can be preserved up to 2–3 weeks at 4°C in water with 0.002–0.01% sodium azide to prevent bacterial growth. Addition of 0.01% sodium azide will reversibly reduce the expansion factor by a factor of ~10%.

### Imaging expanded specimens

The specimen can be immobilized on the bottom of the glass well/plate (optional, for preventing specimen's drift during imaging) with 1.5 % low melting point agarose solution. To do this, carefully add hot 1.5% agarose solution to the perimeter of specimen dropwise until the edge of specimen is covered, then wait for 1 min or until the applied agarose solution solidified. Finally, cover the whole specimen with the rest of agarose solution.

Image the target regions of the expanded tissue by using conventional wide-field or confocal microscopes. Users can choose any objectives desired for their experiments. However, 40 × long-working-distance water immersion objectives are recommended, due to closest matched refractive index, high numeral aperture number and long working distance for large volume imaging with high resolution. Since expansion effectively reduces the thickness of focal plane, we recommend acquiring z stack images of expanded kidney specimen with optimal step size to generate the maximum-intensity projection image if pre- vs. post-expansion comparison of features is needed.

### Image processing

#### Tiling

Imaging large fields of view requires stitching of individual imaged tiles. Direct tiled acquisition is supported by microscopes with motorized stages and corresponding acquisition software, such as NIS Elements from Nikon Instruments, or Zen from Zeiss. Alternatively, images with overlapped regions can also be stitched together using specific software, such as the open-source tools [Image J stitching plug-in or TeraStitcher (14)] or manufacturers' own imaging software.

#### Maximum-intensity projection

By using open-source tools, such as Image J, the final 2D image of the expanded specimen can be obtained by acquiring multiple stack planes covering the thickness of the 3D (expanded specimen) and using Maximum Intensity Projection.

## Expected results and discussion

Human kidney contains an abundance of structural proteins, such as collagen and elastin (15), which posts a challenge to mechanically homogenization and expansion of gelled kidney tissues using earlier versions of ExM protocols, such as the original ExM (13), proExM (16), etc. In this protocol, we overcome the challenge by using the latest method, ExPath (12), which utilizes aggressive proteinase K digestion to eliminate collagens and other structural proteins. The duration of the homogenization step by the proteinase K treatment may vary depending on tissue thickness. For 5 μm thick kidney tissue, 3 h digestion is sufficient for a complete homogenization. Additional digestion will lead to decreased immunofluorescence signals and lower signal-to-noise ratio.

**Figure 5:**
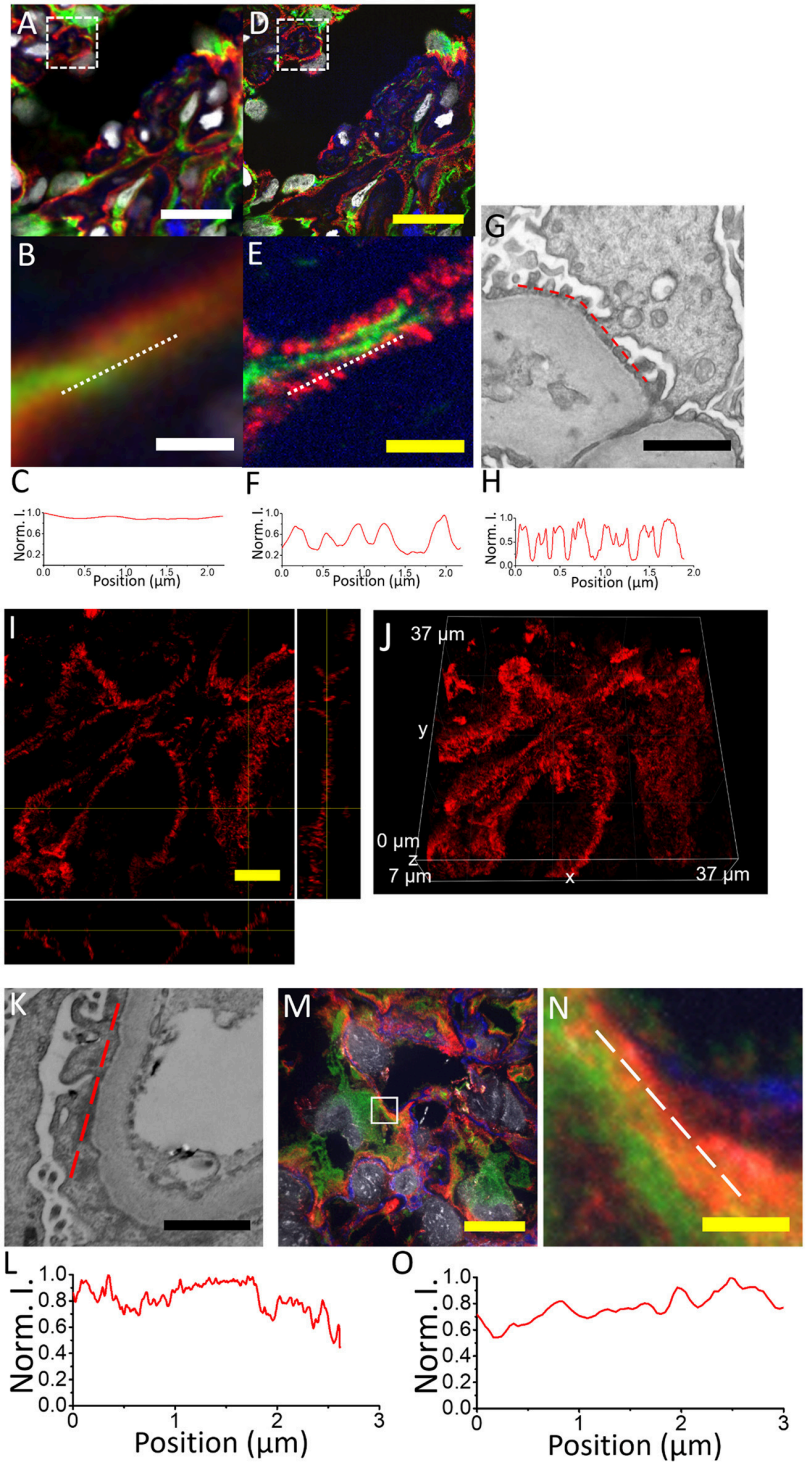
Comparison of kidney specimens pre- vs. post-expansion and post-expansion confocal image of a human kidney sample with minimal change disease acquired with a spinning disk confocal microscope. **(A)** Pre-expansion confocal image of a normal human kidney sample showing part of a glomerulus acquired with a spinning disk confocal microscope. Green, vimentin; red, actinin-4; blue, wheat germ agglutinin; gray, DAPI. Dotted white box shows **(B)**. **(B)** Zoomed-in image of the same kidney specimen using the same microscope. White dotted line indicates the line cut analyzed in **(C)**. **(C)** Profiles of actinin-4 staining intensity along the white dotted lines of **(B)**. **(D)** Post-expansion confocal image of the same normal human kidney sample as in **(A)**, acquired with the same microscope. Green, vimentin; red, actinin-4; blue, wheat germ agglutinin; gray, DAPI. Dotted white box shows **(E)**. **(E)** Zoomed-in image of the same kidney specimen using the same microscope. White dotted line indicates the line cut analyzed in **(F)**. **(F)** Profiles of actinin-4 staining intensity along the white dotted lines of **(E)**. **(G)** Electron micrograph of a normal human kidney sample showing the podocyte tertiary foot processes. **(H)** Profiles of EM contrast along the red dotted lines of **(G)**. **(I)** Orthogonal view of actinin-4 image in part of the expanded kidney glomerulus in **(D)**. **(J)** The 3D image of **(I)**. **(K)** Electron micrograph of a human kidney sample with minimal change disease. **(L)** Profiles of EM contrast along the red dotted lines of **(K)**. **(M)** Post-expansion confocal image of a human kidney sample with minimal change disease acquired with a spinning disk confocal microscope. **(N)** Zoomed-in image of the same kidney specimen as indicated by the white dotted box in **(M)**. Green, vimentin; red, actinin-4; blue, collagen IV; gray, DAPI. **(O)** Profiles of actinin-4 intensity along the white dotted lines of **(N)**. Scale bar: **(A)** 20 μm; **(B)** 1.5 μm; **(D)** 0 μm; (physical size post-expansion, 105 μm; expansion factor, 5.25); **(E)** 1.5 μm (physical size post-expansion, 10.5 μm; expansion factor, 5.25); **(G)** 1 μm; **(I)** 5 μm; (physical size post-expansion, 26.25 μm; expansion factor, 5.25); **(K)** 1 μm; **(M)** 10 μm; physical size post-expansion, 46 μm; expansion factor, 4.6; **(N)** 1 μm; physical size post-expansion, 4.6 μm; expansion factor, 4.6.

Fixation and antigen retrieval steps are critical determinants for high-density staining of the expanded tissue. Both non-aldehyde (e.g., acetone) and aldehyde (e.g., formalin, paraformaldehyde, and glutaraldehyde) fixed kidney tissue specimens can be expanded using this protocol. However, for staining of the podocyte foot processes within the kidney's glomeruli and investigating with precision the ultrafine structure of the tertiary food processes of the podocytes, kidney tissues fixed by non-cross-linking fixatives provide the optimal results (Figure 5). For example, we found two targets (actinin-4 and synaptopodin) and two antibodies (rabbit polyclonal anti-ACTN4 primary antibody from Sigma Aldrich, cat# HPA001873 and mouse monoclonal anti-Synaptopodin from PROGEN Biotechnik, cat# GP94-IN) that provide sufficient labeling density to reveal the nanoscale structure of tertiary podocyte foot processes in acetone-fixed kidney tissues. Using the same set of labeling agents on formalin-fixed specimens (FFPE) have resulted in a punctate staining pattern (12), without a change in expansion efficiency. This difference may potentially be the result of degraded antigenicity caused by formalin-induced cross-linking (12). The punctate staining pattern may lead to misrepresentations of the nanoscale structure of the target. Thus, additional optimization of the staining reagents (e.g., finding the best antibodies/fluorophores) is required for imaging of the tertiary foot processes in FFPE specimens. It is noteworthy that, although the antigen retrieval is usually employed for specimens fixed with an aldehyde fixative (17), our results suggest that the staining of acetone-fixed specimens is also improved after antigen retrieval treatment, resulting in higher quality images after expansion (12).

Imaging of the pre-expansion specimen is recommended. By comparing pre- and post-expansion images, the user can calculate the expansion factor and evaluate the potential distortion after expansion. Expansion factor is determined by the average ratio of the sizes of features measured post-expansion vs. the ones measured before expansion. Around 4.5-fold expansion in pure water is an indication of successful homogenization. The specimen-to-specimen variability of expansion factor is usually within 10% (12, 13, 16). Pre-expansion and post-expansion images of the same field of view should be visually similar. Distortion should be low, typically below 5%.

In Figure 5 we show a comparison of images of the acetone-fixed frozen normal kidney tissue sections acquired pre- vs. post-expansion. We successfully observe the nanoscale anatomy of glomeruli in expanded specimens, revealing ultrafine structures of tertiary podocyte foot processes (Figures 5D–F) not visible in confocal imaging on conventional, un-expanded kidney specimens (Figures 5A–C). The normal kidney tissues present two common features (Figure 5F): (1) The width of podocyte foot processes should be in the range of 200–300 nm (biological scale); (2) Periodic pattern with 200–400 nm gaps in between. If the user possesses a confocal microscopy, volumetric imaging is encouraged and will help validate the results since the features should be consistent in both xz and yz views (Figures 5I,J). In the case of podocyte foot process effacement, these features of normal podocyte foot processes are absent (Figures 5K–O).

The user should note that one apparent limitation of ExPath in comparison with other super-resolution optical imaging techniques is that it is likely not compatible with living specimens. Thus, the use of ExPath on imaging kidney is limited to fixed specimens. Since the expansion process is conducted after conventional immunostaining, it only allows concomitant investigation of up to 4–5 molecular targets with fluorescence microscopy. In addition, imaging the whole expanded tissue slide may take much longer time compared to the conventional IF due to the large increase in specimen volume and dilution of fluorescence signals. For practicality, we recommend quickly scanning the whole tissue using low magnification objectives and then focusing on imaging the regions of interests with higher magnification objectives, which will reduce imaging time and resources. Pre-expansion images will provide guidance for cutting tissue with regions of interest. Moreover, at this time, ExPath does not approximate, or improve the diagnostic accuracy of the EM. While ExPath extends the optical microscopy's resolution limit to about 70 nm, EM can reach a much higher resolution. However, ExPath is a much cheaper, faster and easier method to investigate structures higher than 70 nm (and lower than 300 nm—the resolution limit of an optical microscope) with an optical microscope, after physical tissue expansion, which was not possible before.

ExPath can be applied as an investigational or diagnosis tool in any other kidney pathology where foot process effacement is present. Podocyte foot process effacement can also be found in other proteinuric glomerular diseases, such as the immunoglobulin A nephropathy, and diabetic nephropathy (18), with potential extension to other glomerular-related or unrelated kidney pathologies. For example, ExPath could potentially be used to investigate the molecular markers of mitochondrial biogenesis and function which are modified by sepsis-induced acute kidney injury. Acute kidney injury may result in subtle changes in tubular epithelial mitochondria, which were shown in mouse models to be close indicators of kidney function and recovery (19). While it is outside the scope of this method paper, in the future, large-scale blinded studies using ExPath will be needed to establish the optical diagnosis of nephrotic kidney disease with known nanoscale pathology, such as membrane deposits, inclusions, mitochondrial characteristics.

In conclusion, ExPath enables visualization of tertiary podocyte foot processes in kidney glomeruli using only conventional optical microscopes, which significantly lowers the requirement of equipment and the potential cost for examining kidney biopsies with glomerular diseases, such as MCD and FSGS. In the future, large-scale blinded studies using ExPath and standardization of the protocol may be relevant for streamlining the diagnosis or confirmation of kidney glomerular diseases. This can potentially be extended to other kidney diseases with known nanoscale pathology and early stage diseases with changes that are too subtle to be observed with ordinary microscopes.

## Author contributions

YZ conducted experiments and analyzed data. OB and YZ wrote the text of the article and drew the figures. All authors listed, have made substantial and intellectual contribution to the work, and approved the manuscript.

### Conflict of interest statement

The authors are the inventors in several patents related to Expansion Pathology. OB is the co-Founder and CEO of QPathology LLC, Boston, MA.
